# Early Cardiomyopathy in Prediabetic NDPK-B-Deficient Mice Is Associated with Remodeling of the Mitochondrial O-GlcNAc Proteome

**DOI:** 10.3390/ijms27177518

**Published:** 2026-08-22

**Authors:** Noor Karim, Miao Qin, Rachana Eshwaran, Feng Shao, Santosh Lomada, Merve Keles, Yixin Wang, Felix A. Trogisch, Uwe Schlattner, Joerg Heineke, Thomas Wieland, Yuxi Feng

**Affiliations:** 1 Experimental Pharmacology Mannheim (EPM), European Center for Angioscience, Medical Faculty Mannheim, Heidelberg University, 68167 Mannheim, Germany; noor.karim@medma.uni-heidelberg.de (N.K.); miao.qin@medma.uni-heidelberg.de (M.Q.);; 2 Department of Cardiovascular Physiology, Medical Faculty Mannheim, Heidelberg University, 68167 Mannheim, Germany; 3 Laboratory of Fundamental and Applied Bioenergetics (LBFA), Inserm U1055 and University Grenoble Alpes, 38058 Grenoble, France; uwe.schlattner@univ-grenoble-alpes.fr

**Keywords:** prediabetes, diabetes, NDPK-B, HBP, diabetic cardiomyopathy, mitochondria

## Abstract

Diabetic cardiomyopathy (DCM) is characterized by myocardial remodeling that may already be evident during prediabetes, yet the molecular alterations accompanying these early changes remain poorly understood. The present study examined mouse models of Nucleoside diphosphate kinase B (NDPK-B)-deficient prediabetes and streptozotocin-induced diabetes using O-GlcNAc-associated proteomic profiling to define stage-specific molecular alterations during the progression from prediabetic to diabetic cardiomyopathy. Both models exhibited increased left ventricular extracellular matrix deposition and impaired diastolic function, together with activation of the hexosamine biosynthesis pathway. Profiling of O-GlcNAc-associated proteins uncovered extensive remodeling of the mitochondrial proteome already at the prediabetic stage, with respiratory complex I among the most prominently altered targets, alongside changes in substrate metabolism and inflammatory signaling. In overt DCM, the putative O-GlcNAc proteomic profile was associated with a shift toward wider lipid-dependent metabolic reprogramming and remodeling of mitochondrial proteins. These findings identify early remodeling of the mitochondrial O-GlcNAc-associated proteome as a molecular signature of prediabetic cardiomyopathy and highlight respiratory complex I proteins as candidate targets for future mechanistic investigations.

## 1. Introduction

Diabetic cardiomyopathy (DCM) presents as a progressive decline in myocardial function that begins with an asymptomatic and silent phase [1]. This initial stage is marked by structural and functional hallmarks including increased interstitial accumulation of extracellular matrix (ECM), impaired early diastolic filling, and elevated left ventricular (LV) end-diastolic pressure [2]. Driven centrally by hyperglycemia, the pathophysiology involves aberrant metabolism, cardiac insulin resistance, oxidative stress, and altered growth factor signaling, which collectively advance pathological remodeling. Over time, progressive LV hypertrophy and worsening diastolic dysfunction lead to heart failure with preserved ejection fraction (HFpEF), ultimately transitioning into systolic failure characterized by reduced ejection fraction and ventricular dilation [3]. Notably, significant cardiac alterations in this pathogenic remodeling are already detectable during the prediabetic stage [4].

Under hyperglycemia, the heart shifts towards greater fatty acid (FA) oxidation, while glucose oxidation is suppressed and uncoupled from glycolytic flux. Combined with elevated free FAs, this imbalance overwhelms the mitochondrial capacity, diverting excess FAs into non-oxidative pathways. Accelerated FA oxidation triggers excessive reactive oxygen species (ROS) production, damaging mitochondrial structures, uncoupling oxidative phosphorylation, and impairing ATP production [5]. Despite substantial progress, the precise mechanisms linking altered substrate utilization and mitochondrial dysfunction to contractile impairment in metabolic disorders remain incompletely understood.

Nucleoside diphosphate kinase (NDPK) is a ubiquitous enzyme that catalyzes the transfer of the γ-phosphate group from nucleoside triphosphates (NTPs) to nucleoside diphosphates (NDPs) through a ping-pong mechanism involving a high-energy phosphohistidine intermediate at residue His118 [6,7]. NDPK-B is a member of the NDPK family with a distinct function, serving as a protein histidine kinase interacting with G-protein β-subunits and specific ion channels [8,9]. Recent work identified NDPK-B as a key regulator of endothelial barrier function and angiogenic sprouting, where it mediates Vascular endothelial growth factor (VEGF)-induced disruption of adherens junctions, thereby contributing to vessel outgrowth [10]. Moreover, NDPK-B deficiency not only promotes pericyte loss and subsequent formation of acellular capillaries in the retina but also induces hypertrophy, ECM accumulation and diastolic dysfunction in the heart, resembling the pathology seen in diabetic complications [11,12,13]. Interestingly, retinal vascular damage in NDPK-B-deficient (NDPK-B^−/−^) mice, a model of prediabetes, was further exacerbated by the induction of experimental diabetes. Furthermore, an essential role of endothelial NDPK-B in the development of retinal and cardiac dysfunction has been identified. Endothelial NDPK-B depletion enhances protein O-GlcNAcylation through activation of the hexosamine biosynthetic pathway (HBP) and the subsequent O-GlcNAc cycle [11,12,14,15].

The HBP constitutes a branch of glucose metabolism that normally diverts only a small portion of fructose-6-phosphate away from glycolysis. Its rate-limiting reaction, the conversion of fructose-6-phosphate to glucosamine-6-phosphate, is catalyzed by glutamine: fructose-6-phosphate aminotransferase (GFAT) [16]. The pathway terminates in the formation of UDP-N-acetylglucosamine (UDP-GlcNAc), which is a substrate for O-GlcNAc transferase (OGT), promoting protein O-GlcNAcylation. O-GlcNAcylation is a dynamic and reversible post-translational modification mediated by OGT and reversed by O-GlcNAcase (OGA) [17]. Recent research highlights that NDPK-B deficiency in endothelial cells impairs the function of adjacent cardiomyocytes through paracrine signaling mechanisms. Inhibition of HBP cycling in the endothelium prevents excessive O-GlcNAcylation and subsequently restores cardiomyocyte function. These findings underscore the critical role of endothelial HBP as a mediator in the crosstalk between the vasculature and the myocardium [12].

Building on our previous data showing that (NDPK-B^−/−^) mice exhibit a prediabetic phenotype linked to HBP activation in the retina and left ventricle, this study aims to delineate the stage-dependent progression of DCM. Specifically, we compared the cardiac phenotypes of prediabetic NDPK-B KO hearts and Streptozotocin (STZ)-induced diabetic hearts. Concurrently, we utilized succinylated wheat germ agglutinin (sWGA)-enriched proteomics to uncover the stage-dependent molecular mechanisms linked to aberrant O-GlcNAc-associated protein alterations in the heart.

## 2. Results

### 2.1. Diabetes Is Successfully Induced and NDPK-B Deficiency Increases Relative Heart Weight in Mice

**Figure 1:**
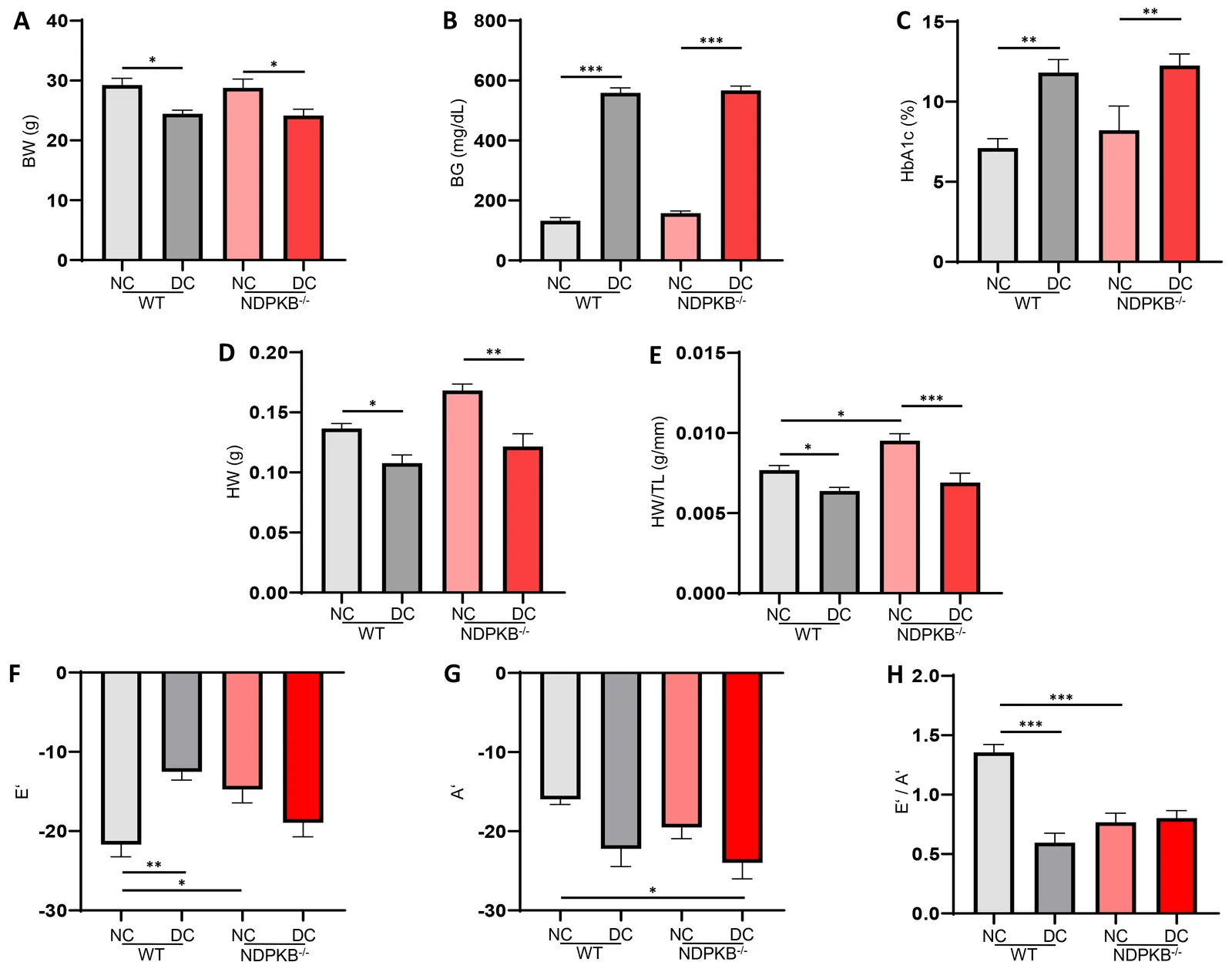
Diabetic and prediabetic NDPK-B^−/−^ mice show diastolic dysfunction. General parameters and echocardiographic characteristics of WT and NDPK-B^−/−^ mice were analyzed 3 months after STZ injection. Quantification of (**A**) BW, (**B**) BG, (**C**) HbA1c, (**D**) HW, (**E**) HW/TL, (**F**) E′, (**G**) A′, (**H**) E′/A′. WT: wild-type; NDPK-B^−/−^: NDPK-B-deficient; NC: non-diabetic; DC: diabetic; STZ: streptozotocin; BW: bodyweight; BG: blood glucose; HbA1c: glycated hemoglobin; HW: heart weight; TL: tibia length; E′: early diastolic annular velocity; A′: late diastolic annular velocity. n = 8–10 for general parameters. n = 6–8 for echocardiography. * *p* < 0.05; ** *p* < 0.01; *** *p* < 0.001.

Key general metabolic parameters were analyzed to confirm successful diabetes induction and to assess genotype- and diabetes-dependent effects. Loss of body weight is a characteristic feature of type 1 diabetes. In line with this, both diabetic (DC) wild-type (WT) and diabetic NDPK-B^−/−^ mice exhibited a significant reduction in body weight compared with non-diabetic (NC) counterparts (Figure 1A, *p* < 0.05).

As shown in Figure 1B, random blood glucose levels were significantly increased in diabetic WT mice compared with their non-diabetic controls (Figure 1B, *p* < 0.001). A comparable increase in blood glucose was also observed in diabetic NDPK-B^−/−^ mice compared with normoglycemic NDPK-B^−/−^ mice (Figure 1B, *p* < 0.001). Consistent with random blood glucose measurements, HbA1c levels were significantly elevated in both diabetic WT and NDPK-B^−/−^ mice compared with their respective non-diabetic controls (Figure 1C, WTNC vs. WTDC: *p* < 0.01; NDPK-B^−/−^NC vs. NDPK-B^−/−^DC: *p* < 0.01).

To evaluate the influence of genotype and glycemic status on cardiac mass, absolute heart weight (HW) was assessed across all experimental groups. The induction of diabetes resulted in a significant reduction in absolute HW in mice compared with normoglycemic controls (Figure 1D, *p* < 0.05). Under normoglycemic conditions, NDPK-B^−/−^ mice exhibited a trend towards increased absolute HW compared with WTNC animals (Figure 1D, *p* > 0.05). Consistent with the observations in the WT group, hyperglycemia in the NDPK-B^−/−^ cohort led to a significant decrease in absolute heart weight in comparison with their normoglycemic counterparts (Figure 1D, *p* < 0.01). When HW was normalized to bodyweight (BW), diabetic WT mice demonstrated values comparable to those of non-diabetic controls (Supplementary Figure S1).

When HW was normalized to tibial length (TL), non-diabetic NDPK-B^−/−^ mice showed a significantly higher relative HW compared with WT controls (Figure 1E, *p* < 0.05). Induction of diabetes led to a significant reduction in relative heart weight in both WT and NDPK-B^−/−^ mice compared with their respective non-diabetic controls. In NDPK-B^−/−^ mice, hyperglycemia markedly reduced the HW/TL ratio (Figure 1E, *p* < 0.05), resulting in values comparable to those observed in diabetic WT mice. These results suggest that diabetes-induced cardiac mass loss overrides the genotype-dependent increase in heart weight observed under normoglycemic conditions.

### 2.2. Both Diabetic and Prediabetic NDPK-B-Deficient Mice Exhibit Diastolic Dysfunction

To evaluate alterations in cardiac function, transthoracic echocardiography was performed, with LV morphology and systolic performance assessed via M-mode. The average heart rate remained comparable among the groups at approximately 400 beats per minute (bpm) (WTNC: 402.37 ± 52.97 bpm, WTDC: 379.87 ± 48.86 bpm, NDPK-B^−/−^NC: 397.28 ± 45.10 bpm, NDPK-B^−/−^DC: 412.52 ± 72.77 bpm). No significant differences were observed among the groups regarding LV structural parameters or global systolic indices, indicating that systolic function remained preserved across all experimental conditions. However, PW Doppler analysis revealed altered diastolic filling, with a significantly reduced E wave in diabetic WT mice compared with non-diabetic WT controls (WTNC: 543.82 ± 74.27 vs. WTDC: 421.35 ± 75.69, *p* < 0.05), whereas the A wave and the E/A ratio remained unaltered. Tissue Doppler imaging further supported these diastolic abnormalities. E′, a sensitive marker of myocardial relaxation, was markedly reduced in both diabetic WT mice and non-diabetic NDPK-B^−/−^ mice compared with WT controls (Figure 1F, WTDC vs. WTNC, *p* < 0.01; NDPK-B^−/−^NC vs. WTNC, *p* < 0.05). Furthermore, A′ was significantly increased only in diabetic NDPK-B^−/−^ mice compared with WT controls. The E′/A′ ratios were significantly reduced in all experimental groups compared with WT non-diabetic controls (Figure 1H, *p* < 0.001 for all comparisons to WTNC), confirming impaired diastolic function under hyperglycemic and NDPK-B-deficient conditions.

### 2.3. Cardiac Fibrosis in Left Ventricles Is Increased Under Diabetic Conditions and Exacerbated by NDPK-B Deficiency

**Figure 2:**
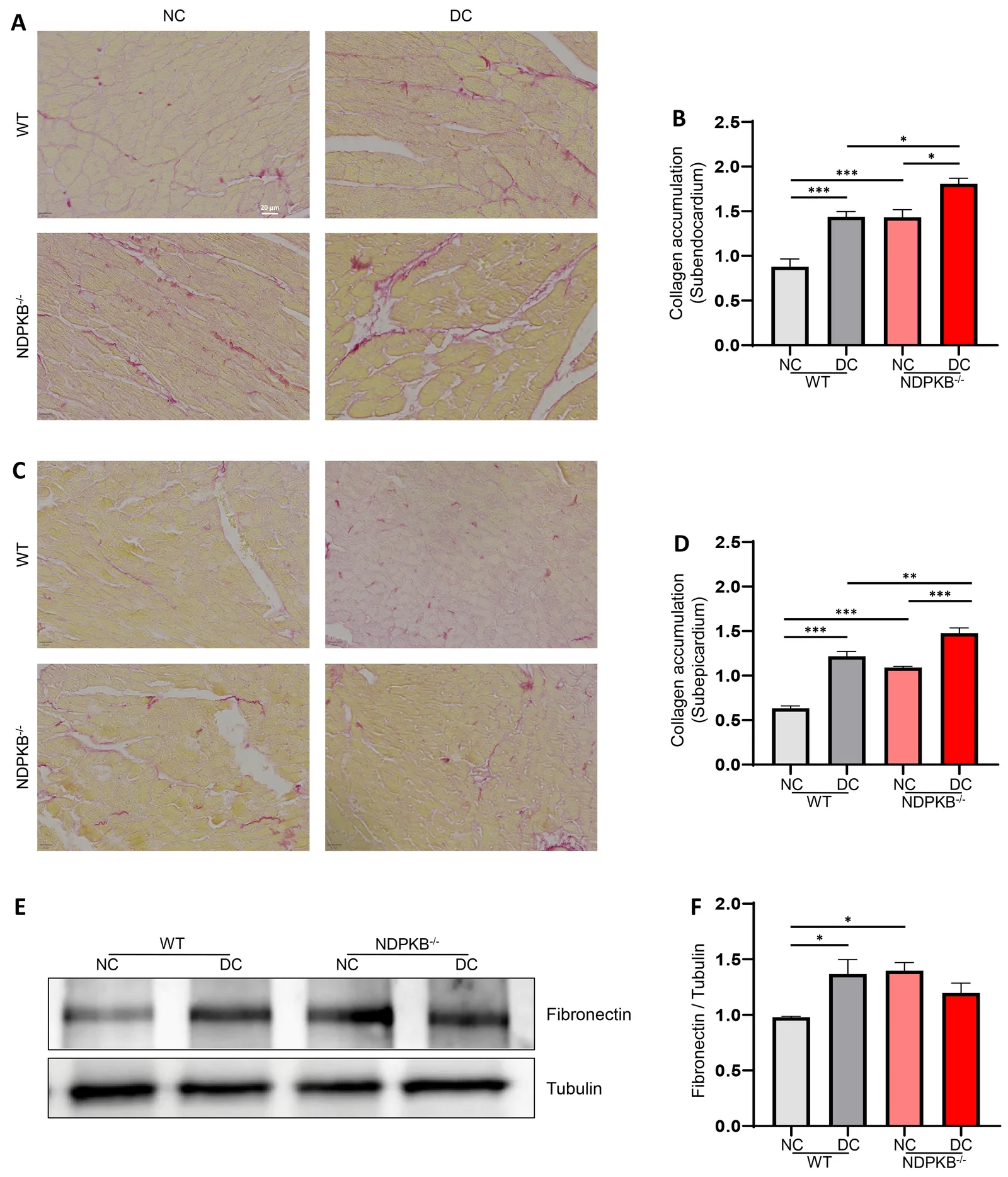
Accumulation of ECM in diabetic and prediabetic NDPK-B^−/−^ LVs. Collagen accumulation and fibronectin protein abundance in LVs. (**A**) Representative images of the subendocardial region stained with Sirius Red. (**B**) Quantification of ECM accumulation in the subendocardial region. (**C**) Representative images of the subepicardial region stained with Sirius Red. (**D**) Quantification of ECM accumulation in the subepicardial region. (**E**) Representative Western blots showing fibronectin protein abundance. (**F**) Quantification of fibronectin protein abundance. WT: wild-type; NDPK-B^−/−^: NDPK-B-deficient; NC: non-diabetic; DC: diabetic; LV: left ventricle; ECM: extracellular matrix. n = 5–8, * *p* < 0.05; ** *p* < 0.01; *** *p* < 0.001.

Given the alterations in heart weight and diastolic dynamics, we next investigated ECM accumulation by assessing collagen accumulation (Figure 2A,C). Compared with non-diabetic controls, diabetic WT LVs exhibited a significant increase in collagen deposition within the subendocardial layer (Figure 2B, *p* < 0.001). Even under normoglycemic conditions, NDPK-B^−/−^ LVs displayed substantially elevated subendocardial ECM accumulation compared with WTs (Figure 2B, *p* < 0.001). Interestingly, diabetic NDPK-B^−/−^ LVs showed the highest level of subendocardial collagen deposition among all experimental groups (*p* < 0.05). Regional analysis further confirmed similar results in the subepicardial layer (Figure 2D).

To further characterize ECM remodelling, fibronectin abundance was assessed through immunoblot analysis of LV protein lysates (Figure 2E). WTDC LVs revealed elevated fibronectin levels compared with WTNC LVs. NDPK-B^−/−^ LVs displayed fibronectin abundance comparable to WTDC levels. Interestingly, diabetes did not further augment fibronectin accumulation in NDPK-B^−/−^ LVs (Figure 2F). Increased fibronectin abundance in hyperglycemic WTs and normoglycemic NDPK-B^−/−^ LVs further supports enhanced ECM accumulation.

### 2.4. Prediabetic NDPK-B Deficiency but Not STZ-Induced Diabetes Enhances Cardiomyocyte Hypertrophy in the Left Ventricle

**Figure 3:**
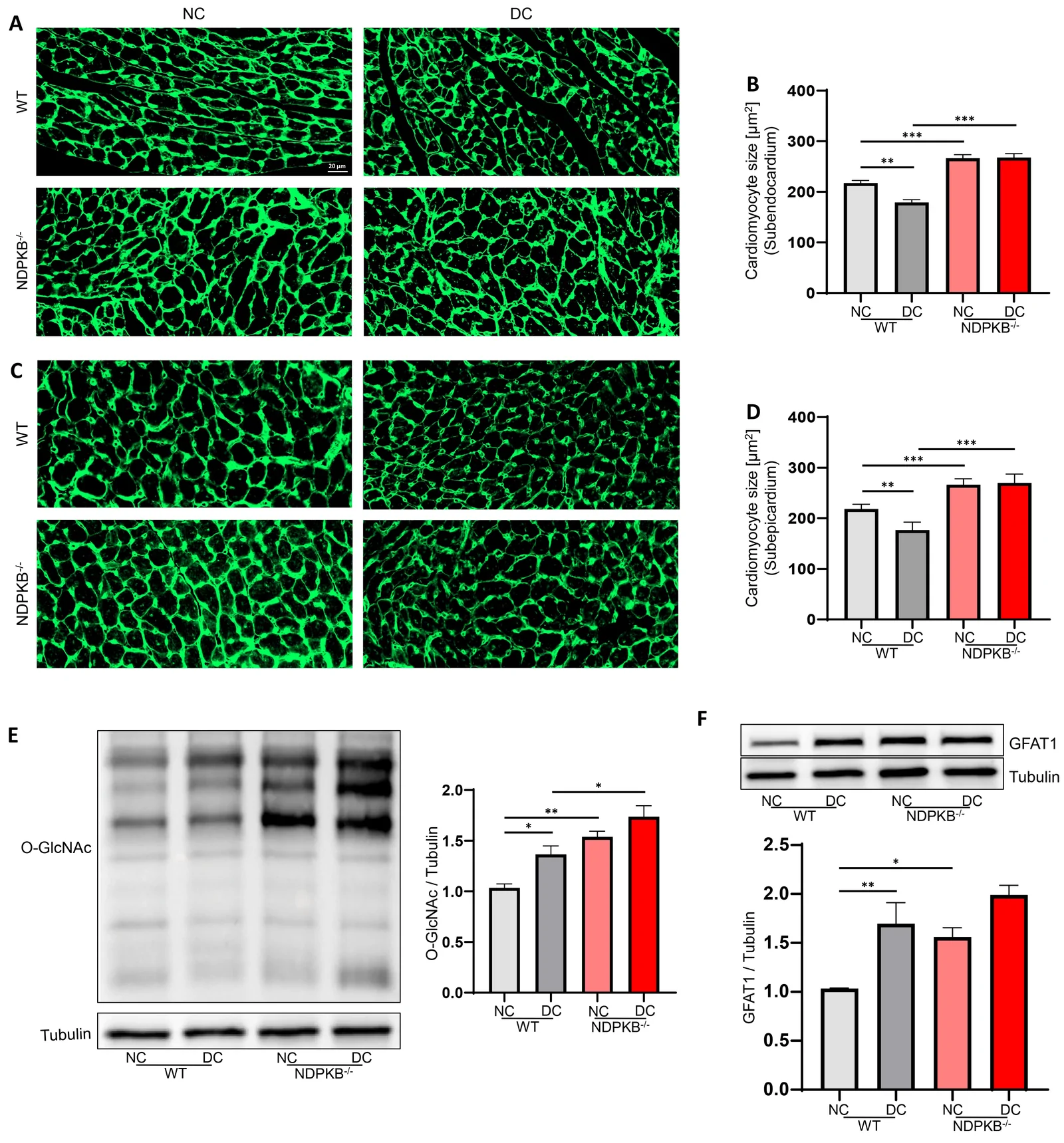
Cardiac hypertrophy and increased O-GlcNAcylation in diabetic and prediabetic NDPK-B^−/−^ LVs. Cell size was assessed by WGA staining of LV sections, and global protein O-GlcNAcylation and GFAT1 abundance were determined by immunoblot analysis. (**A**) WGA-stained images. (**B**) Quantification of cell size in the subendocardial region. (**C**) Representative WGA-stained images of the subepicardial region stained with WGA. (**D**) Quantification of cell size in the subepicardial region. (**E**) Representative immunoblots illustrating global O-GlcNAc protein modification and densitometric quantification of global protein O-GlcNAcylation normalized to γ-tubulin. (**F**) Representative immunoblots illustrating GFAT1 protein abundance and densitometric quantification of GFAT1 normalized to γ-tubulin. WT: wild-type; NDPK-B^−/−^: NDPK-B-deficient; NC: non-diabetic; DC: diabetic; WGA: wheat germ agglutinin; LV: left ventricle; GFAT1: glutamine-fructose-6-phosphate aminotransferase 1. n = 5. * *p* < 0.05; ** *p* < 0.01; *** *p* < 0.001.

To investigate whether NDPK-B deficiency contributes to cardiac hypertrophy, cardiomyocyte cross-sectional areas were quantified in LV sections using wheat germ agglutinin (WGA) staining (Figure 3A,C). Regional analysis of the subendocardium revealed a significant reduction in cardiomyocyte size in diabetic WTs compared with non-diabetic controls (Figure 3B, *p* < 0.01). This is consistent with the previously reported reduction in heart weight in hyperglycemic mice. In contrast, NDPK-B^−/−^ LVs displayed a marked increase in cardiomyocyte size in the subendocardial region, aligning with the described increase in relative heart weight (Figure 3B, *p* < 0.001). Importantly, although diabetic NDPK-B^−/−^ mice exhibited significant reductions in both body weight and heart weight compared with non-diabetic NDPK-B^−/−^ mice, cardiomyocyte sizes in the LV remained unchanged between these two groups. Analysis of the subepicardial region revealed a similar pattern to that observed in the subendocardium, with NDPK-B deficiency consistently associated with enlarged cardiomyocytes regardless of diabetic status (Figure 3D). These data suggest that NDPK-B deficiency is associated with LV cardiomyocyte hypertrophy independently of diabetes or overall HW.

### 2.5. The Hexosamine Biosynthetic Pathway Is Activated in Diabetic and Prediabetic NDPK-B-Deficient Left Ventricles

Given the established role of the HBP in diabetes, we examined whether HBP activity is altered under diabetic conditions or by the loss of NDPK-B in LVs. Consistent with observations in diabetic WT LVs, LVs from NDPK-B^−/−^ mice exhibited markedly elevated levels of protein O-GlcNAcylation. Importantly, this genotypic increase was also evident under diabetic conditions, with hyperglycemic NDPK-B^−/−^ LVs displaying significantly higher global O-GlcNAc levels compared with diabetic WT LVs (Figure 3E).

To further investigate the mechanisms underlying increased O-GlcNAc signaling, we examined the abundance of the key enzymes responsible for O-GlcNAc cycling. Interestingly, no significant changes were observed in the protein abundance levels of OGT or OGA across any of the experimental groups (Supplementary Figure S2A,B). Instead, we found that the protein abundance of GFAT1 was significantly increased in both diabetic and NDPK-B^−/−^ LVs (Figure 3F).

### 2.6. Aberrant Putative O-GlcNAcylation of Mitochondrial Proteins Is Associated with the Early Pathological Changes Preceding Diabetic Cardiomyopathy

**Figure 4:**
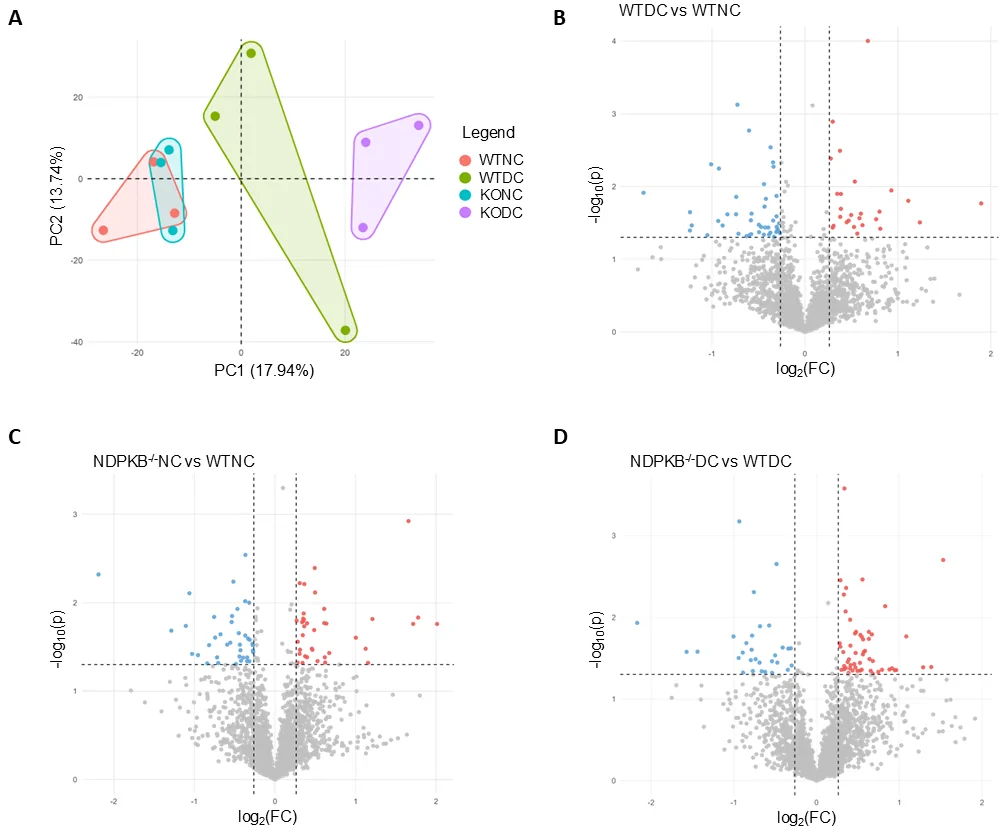
Distinct clusters of sWGA-enriched protein profiles in the volcano plots of diabetic and prediabetic NDPK-B^−/−^ LVs. Proteomic analysis was performed on putative O-GlcNAcylated proteins enriched from left ventricular tissue using sWGA. (**A**) Principal component analysis showing clustering of WTNC, WTDC, NDPK-B^−/−^NC, and NDPK-B^−/−^DC samples. (**B**) Volcano plot of WTDC versus WTNC. (**C**) Volcano plot of NDPK-B^−/−^NC versus WTNC. (**D**) Volcano plot of NDPK-B^−/−^DC versus WTDC. In volcano plots, the *x*-axis represents the log2 fold change (log_2_FC) and the *y*-axis represents the *p*-value (−log_10_(*p*)). Dotted lines indicate significance and fold-change thresholds. Red dots indicate significantly increased enrichment, whereas blue dots indicate significantly decreased enrichment compared with the respective control group. Grey dots represent non-significant changes. WT: wild-type; NDPK-B^−/−^: NDPK-B-deficient; NC: non-diabetic; DC: diabetic; sWGA: succinylated wheat germ agglutinin; LV: left ventricle; FC: fold change; PCA: Principal Component Analysis; PC: Principal Component. n = 3.

Given that activation of the HBP is a common upstream mechanism shared by prediabetes and diabetes, we sought to identify stage- and HBP-dependent proteins and pathways involved in the pathological process. O-GlcNAc proteins represent a relatively small and low-abundance subset of the proteome. For our purpose, O-GlcNAc-associated proteins from LVs were enriched by immunoprecipitation using sWGA and subsequently analyzed by proteomics. Principal Component Analysis (PCA) of the sWGA-enriched LV proteomic profiles revealed a clear separation of samples primarily driven by diabetic status along PC1, which accounted for 17.94% of the total variance, and PC2, which mainly reflected inter-sample variability and accounted for 13.74%. WTDC samples were distinctly separated from WTNC along PC1, indicating a dominant diabetes-induced remodeling of the O-GlcNAc-enriched proteome. In contrast, NDPK-B^−/−^ samples exhibited a markedly different clustering behavior. NDPK-B^−/−^NC replicates were already displaced from WTNC toward the diabetes-associated PC1 axis, suggesting that NDPK-B deficiency alone induces a basal sWGA-enriched protein profile resembling diabetic conditions. Moreover, NDPK-B^−/−^DC samples clustered further away from WTDC, indicating that hyperglycemia exacerbates O-GlcNAc-associated remodeling in NDPK-B-deficient conditions (Figure 4A).

Volcano plot analyses further substantiated these observations by revealing both diabetes- and genotype-specific changes in the sWGA-enriched proteome. In WT mice, diabetes resulted in a moderate but significant remodeling of the LV O-GlcNAc-enriched proteome, with increased enrichment observed in 26 proteins and decreased modification in 44 proteins compared with WTNC controls (Figure 4B). Similarly, NDPK-B deficiency under normoglycemic conditions resulted in substantial baseline differences compared with WTNC, with increased enrichment in 39 proteins and decreased enrichment in 40 proteins (Figure 4C). Under diabetic conditions, however, genotype differences became more pronounced, with NDPK-B^−/−^DC LVs exhibiting increased enrichment in 55 proteins and decreased enrichment in 30 proteins compared with WTDC (Figure 4D). Significantly enriched proteins have been summarized in Supplementary Table S1. These data demonstrate that NDPK-B deficiency alone elicits alterations comparable to those induced by diabetes. Notably, the combination of NDPK-B deficiency and diabetes resulted in a distinct and amplified O-GlcNAc-associated proteomic signature.

**Figure 5:**
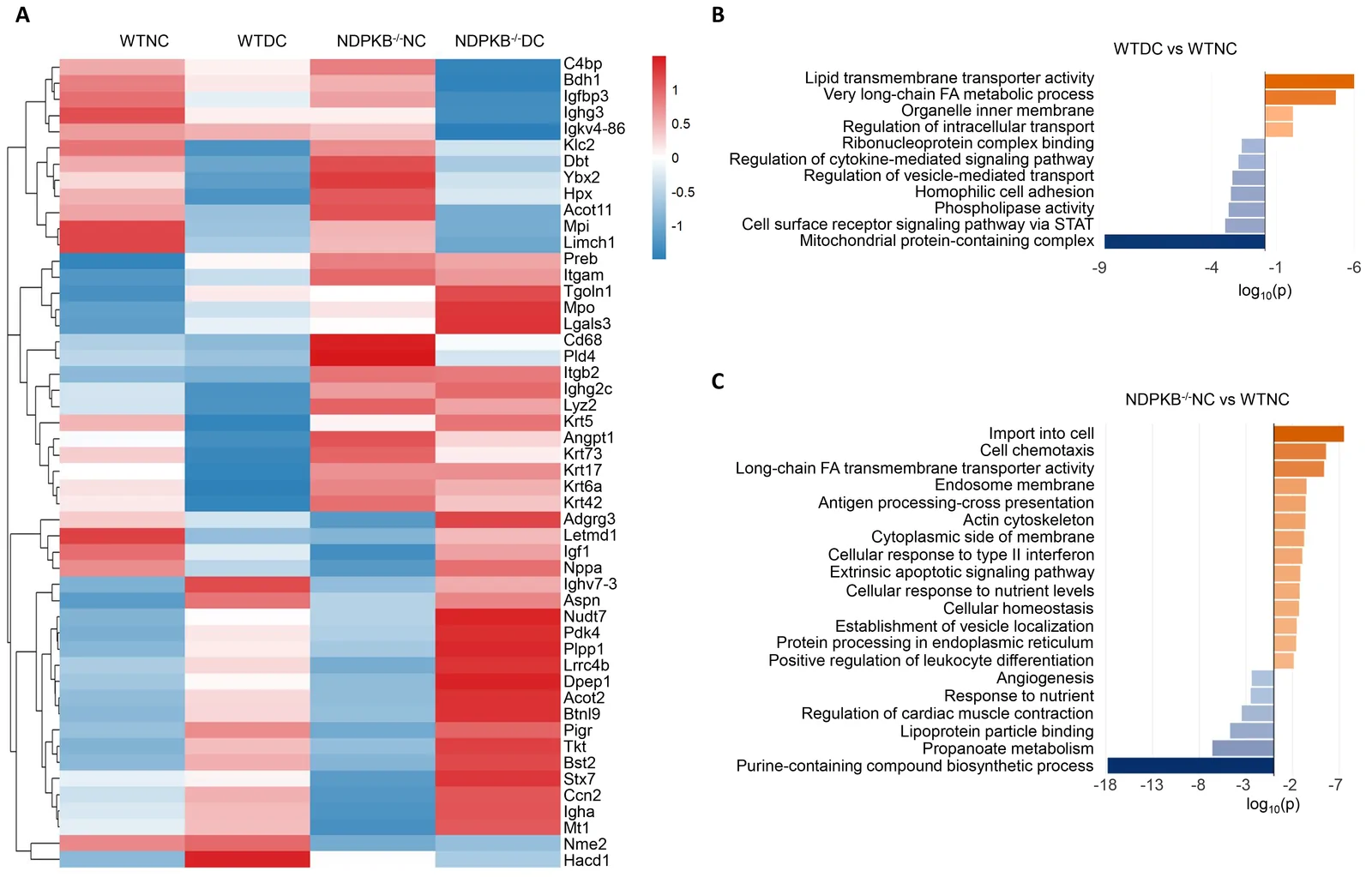
Stage-specific alterations define distinct O-GlcNAc-associated protein profiles in prediabetic and diabetic cardiomyopathy. Hierarchical clustering and pathway enrichment analysis were performed based on significantly altered sWGA-enriched proteins identified in LVs. (**A**) Heatmap representing the top 50 altered proteins. Colors indicate row-scaled Z-scores of protein abundances (red: increased; blue: decreased). (**B**) Enriched pathways in WTDC versus WTNC. Bars represent significantly enriched GO and KEGG terms plotted as log_10_(*p*). (**C**) Enriched pathways in NDPK-B^−/−^NC versus WTNC plotted as log_10_(*p*). WT: wild-type; NDPK-B^−/−^: NDPK-B-deficient; NC: non-diabetic; DC: diabetic; sWGA: succinylated wheat germ agglutinin; LV: left ventricle.

To visualize the most pronounced alterations within the sWGA-enriched proteome, we generated a heatmap representing the top 50 differentially regulated proteins (Figure 5A). Hierarchical clustering revealed distinct, genotype- and disease-specific patterns. A specific subset of proteins (PREB, ITGAM, TGOLN1, MPO, LGALS3, CD68, PLD4, ITGB2, IGHG2C, LYZ2, KRT5, ANGPT1, KRT73, KRT17, KRT6A, KRT42) exhibited low relative enrichment in WT LVs but increased upon NDPK-B deficiency. Furthermore, gene annotation analysis identified a robust association between these proteins and inflammatory signaling, hinting at a regulatory role for NDPK-B in inflammatory pathways. The diabetic condition in LVs was characterized by the elevated enrichment of a distinct cluster, encompassing IGHV7-3, ASPN, NUDT7, PDK4, PLPP1, LRRC4B, DPEP1, ACOT2, BTNL9, PIGR, TKT, BST2, STX7, CCN2, IGHA, and MT1. This elevation was further exacerbated in the NDPK-B^−/−^DC group. Modification levels for these proteins were profoundly lower in both NDPK-B^−/−^NC and WTNC LVs, identifying this as a disease-specific pattern. Conversely, an inverse cluster comprising KLC2, DBT, YBX2, HPX, ACOT11, MPI, and LIMCH1 was identified. Furthermore, another specific subset of proteins (ADGRG3, LETMD1, IGF1, NPPA) showed a moderate decrease in WTDC LVs, mirroring the changes observed in the NDPK-B^−/−^NC group and suggesting shared regulatory pathways.

To determine commonly affected proteins and pathways in prediabetic NDPK-B^−/−^ LVs and diabetic LVs, we systematically compared their profiles. For this purpose, two essential regulatory patterns were defined. This comparative analysis enabled the organization of proteins into distinct regulatory patterns reflecting common or condition-specific mechanisms. Notably, five proteins (RAB5A, MAP2K2, TAGLN2, LETM1, and PDIA3) displayed significantly increased enrichment in both WTDC and NDPK-B^−/−^NC LVs relative to WTNC LVs, defining pattern 1. This shared enhancement suggests that these proteins are consistent targets of HBP activation in both NDPK-B deficiency and diabetes-driven metabolic stress. Conversely, two proteins (NRCAM and CDH13) exhibited reduced enrichment in both WTDC and NDPK-B^−/−^NC LVs compared with WTNC LVs (pattern 2). The regulation profiles of patterns 1 and 2 were summarized in Supplementary Table S1 and visualized in Supplementary Figure S3A. Pathway annotation analysis of these seven overlapping proteins revealed a significant enrichment in cell–cell adhesion, regulation of vesicle-mediated transport, and membrane organization (Supplementary Figure S3B). Given that the NDPK-B-deficient mouse model represents a prediabetes-like state, these overlapping signatures delineate molecular changes shared by early and overt cardiomyopathy.

To better understand the distinct mechanisms involved in biological functions during the initiation and progression of DCM, we conducted GO functional and KEGG pathway enrichment analyses among the control and NDPK-B^−/−^ prediabetic and diabetic groups. Overt DCM was associated with altered enrichment of proteins, primarily linked to increased lipid transmembrane transporter activity and very long-chain FA metabolic processes. In parallel, significant downregulation of mitochondrial protein-containing complexes was observed (Figure 5B). In contrast, the NDPK-B-deficient prediabetic stage demonstrated markedly decreased enrichment associated with the purine-containing compound biosynthetic process and increased enrichment of proteins associated with pathways related to cellular import, chemotaxis, and long-chain FA transmembrane transporter activity (Figure 5C).

**Figure 6:**
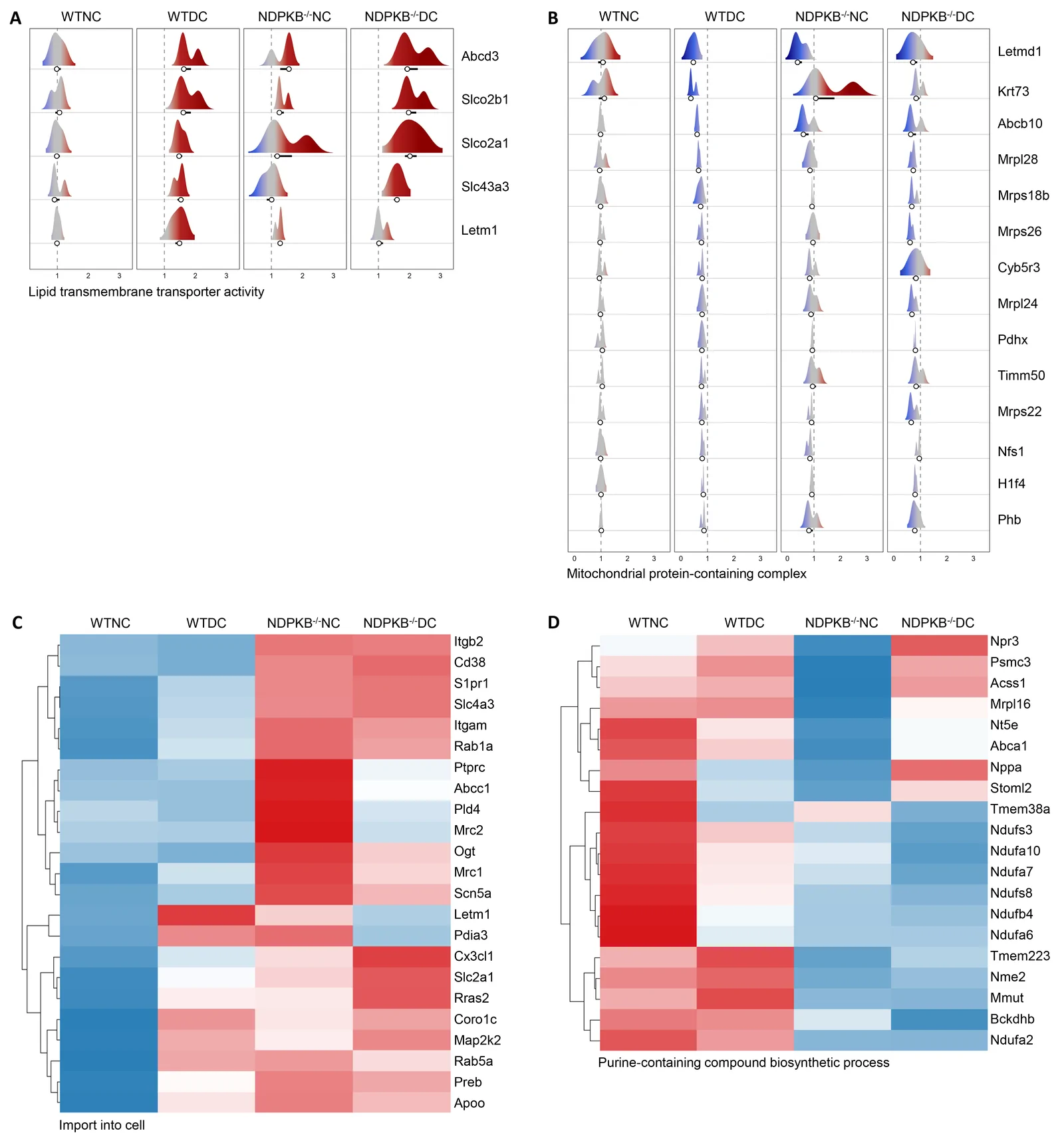
Prediabetic and diabetic cardiomyopathy drive pathway-specific regulation of target proteins. Violin plots illustrating the distribution of fold changes for proteins within significantly enriched pathways. The *x*-axis and color scale represent the direction and magnitude of regulation (red: upregulated; blue: downregulated), while the width of each violin reflects the density of the distribution. (**A**) Proteins associated with lipid transmembrane transporter activity, exclusively showing upregulated candidates in WTDC versus WTNC. (**B**) Proteins within the mitochondrial protein-containing complex, representing exclusively downregulated candidates in WTDC versus WTNC. Hierarchical clustering and heatmaps showing row-scaled Z-scores of protein abundances (red: increased; blue: decreased). (**C**) Proteins involved in import into cells, displaying upregulated proteins in NDPK-B^−/−^NC compared with WTNC. (**D**) Proteins related to the purine-containing compound biosynthetic process, depicting downregulated proteins in NDPK-B^−/−^NC versus WTNC. WT: wild-type; NDPK-B^−/−^: NDPK-B-deficient; NC: non-diabetic; DC: diabetic.

Detailed examination of the top regulated pathways in the WTDC versus WTNC and NDPK-B^−/−^NC versus WTNC comparisons suggested distinct molecular patterns. The majority of proteins associated with lipid transmembrane transporter activity that demonstrated increased enrichment in WTDC showed further enrichment in the NDPK-B^−/−^DC group (Figure 6A). A comparable pattern emerged for proteins within the mitochondrial protein-containing complex, which showed decreased enrichment in WTDC and displayed further suppression in NDPK-B^−/−^DC (Figure 6B). Interestingly, several target proteins exhibited similar relative alterations in both the WTDC and NDPK-B^−/−^NC groups. Violin plot analysis also revealed a narrow distribution of protein enrichment in Figure 6B within the WTDC group.

Furthermore, the data revealed a distinct sWGA-enriched protein pattern in prediabetic NDPK-B^−/−^ LVs. The majority of proteins involved in cell import dynamics, specifically those mediating glucose, fatty acid, amino acid, and ion uptake, were elevated in the NDPK-B^−/−^NC group (Figure 6C). In contrast, proteins associated with purine-containing compound biosynthetic processes were distinctly downregulated in NDPK-B^−/−^NC LVs. Notably, within this subset, seven Ndufa proteins, which are structural components of complex I, were downregulated in a strictly genotype-specific manner (Figure 6D). Moreover, the alteration may be accompanied by increased immune activity and an inflammatory response, as evidenced by increased O-GlcNAc-associated modification of CORO1C, ITGAM, ITGB2, MRC1, PLD4, PTPRC, and CX3CL1. The mitochondrial protein LETM1, which regulates calcium levels and energy generation, exhibited elevated putative O-GlcNAcylation. Similarly, increased modification was detected for the endoplasmic reticulum (ER)-associated proteins PREB and PDIA3.

## 3. Discussion

In the present study, we compared prediabetic NDPK-B^−/−^ LVs with STZ-induced diabetic LVs. We found that cardiomyopathy in prediabetic NDPK-B^−/−^ mice recapitulates several key pathological features of DCM, including cardiac diastolic dysfunction, excessive accumulation of ECM, and activation of the HBP. Importantly, this HBP activation, driven by metabolic dysregulation, is likely to initiate cardiomyopathy primarily through early alterations in O-GlcNAc-associated mitochondrial proteins.

This study established a substantial overlap in cardiomyopathic features between prediabetic NDPK-B^−/−^ hearts and STZ-induced DCM. Notably, despite the absence of overt hyperglycemia, prediabetic NDPK-B^−/−^ mice exhibited pathological activation of the HBP and developed cardiac dysfunction that closely resembles DCM. The activation of the HBP can be at least partly explained by an increased abundance of GFAT1 in the tissue. This elevates the intracellular UDP-GlcNAc pool, ultimately resulting in increased global O-GlcNAcylation. These findings support the suitability of NDPK-B deficiency as a model for investigating early mechanisms underlying metabolically associated cardiomyopathy, particularly those that precede systemic metabolic derangements. These findings are consistent with the report by Shao et al., who reported impaired glucose tolerance in NDPK-B^−/−^ mice [12], suggesting that loss of NDPK-B perturbs metabolic regulatory networks that significantly overlap with prediabetic pathology.

A notable divergence from classical DCM observed in the present study is the absence of cardiac hypertrophy in STZ-treated WT mice. This finding may represent an inherent limitation of the STZ model, as multiple studies have reported reduced heart size in STZ-induced diabetes compared with non-diabetic controls [18,19,20]. The mechanisms underlying this discrepancy remain incompletely understood. In particular, it is unclear whether O-GlcNAcylation and related metabolic signaling pathways are less directly involved in hypertrophic remodeling than in the development of functional impairment and myocardial fibrosis. The apparent reduction in absolute heart weight and cell size in these animals could presumably be a secondary consequence of the systemic weight loss typically associated with STZ-induced toxicity, rather than a primary cardiac defect. Nonetheless, this distinction warrants further investigation, as it may help refine our understanding of the stage- and context-dependent effects of metabolic stress on the heart. Nevertheless, STZ-induced DCM retains key pathological hallmarks of diabetic heart disease, including diastolic dysfunction and ECM remodeling, despite a reduction in overall cardiac mass. In contrast, prediabetic NDPK-B^−/−^ mice exhibited increased relative heart weight under normoglycemic conditions, indicating the presence of intrinsic cardiac pathology that is independent of systemic hyperglycemia. This observation suggests that NDPK-B deficiency likely promotes hypertrophic remodeling through mechanisms similar to those seen in human DCM [21,22]. Together, these findings reinforce the concept that disrupted metabolic signaling can act as a primary driver of cardiomyopathy, initiating structural and functional abnormalities prior to the development of overt diabetes.

The main finding of this study is that prediabetic cardiomyopathy is likely initiated through alterations of the mitochondrial O-GlcNAc-associated proteome. Given the shared dysregulation of O-GlcNAc-associated proteins in conditions of prediabetic NDPK-B deficiency and diabetes, proteomic analysis of NDPK-B^−/−^ LVs provides important mechanistic insights into the early stage of DCM. These mechanisms may be particularly relevant to patients with glucose intolerance, obesity, or early metabolic disorders. Consistent with previous studies, our proteomic analysis revealed an association between HBP activation and alterations in mitochondrial proteins in DCM [23,24,25]. Compared with control LVs, prediabetic NDPK-B^−/−^ LVs displayed a mixed pattern of unchanged, increased, and decreased protein enrichment. This observation aligns with reports by Ma and Banerjee, which describe bidirectional regulation of protein O-GlcNAcylation in rodent hearts under metabolic stress [26,27], highlighting the dynamic and context-dependent nature of this post-translational modification.

Further crucial insights were obtained from volcano plot analysis, GO enrichment, and KEGG pathway assessment. Proteins showing increased enrichment in prediabetic NDPK-B^−/−^ LVs were predominantly involved in pathways related to cellular import, chemotaxis, and long-chain FA transmembrane transporter activity, whereas proteins associated with the purine-containing compound biosynthetic process, including mitochondrial complex I subunits, were significantly downregulated. This latter category included seven NDUF proteins, which are essential subunits of mitochondrial complex I within the electron transport chain. The coordinated downregulation of these proteins strongly suggests a reduction in mitochondrial oxidative phosphorylation and energy production. Mitochondrial complex I impairment has been well documented as a key contributor to the development of diabetic complications [28,29,30,31]. Extending these observations beyond classical diabetic pathology, a recent study demonstrated that under metabolic stress induced by a high-fat diet, mitochondrial function is compromised through a consistent reduction in complex I NDUF protein O-GlcNAcylation [32]. These findings closely parallel the observations reported here and support the concept that alterations in O-GlcNAc-associated Complex I components represent an early and critical driver of mitochondrial remodeling. In addition, our data suggest that mitochondrial remodeling is accompanied by broader metabolic remodeling, reflected in a distinct protein signature indicative of impaired processing of multiple energy substrates, including glucose, fats, and amino acids. This is evidenced by significant alterations in the enrichment of key metabolic enzymes and transporters such as MMUT, BCKDHB, ABCA1, and ACSS1. Together, these changes point toward a metabolic reprogramming response in the prediabetic heart. Interestingly, the cardiac tissue appears to undergo compensatory adaptation to these mitochondrial alterations. This response is marked by increased enrichment of O-GlcNAc-associated proteins involved in immune and inflammatory pathways (CORO1C, ITGAM, ITGB2, MRC1, PLD4, PTPRC, CX3CL1), enhanced cellular fuel uptake (SLC2A1/GLUT1, ABCC1), and vesicle trafficking disruption reflected by Rab protein accumulation. Moreover, increased enrichment of ER-associated proteins such as PREB and PDIA3 suggests substantial ER stress. The simultaneous downregulation of mitochondrial energy-producing proteins and upregulation of proteins involved in cellular import and inflammatory pathways in NDPK-B^−/−^ hearts hint at a compensatory yet ultimately pathological shift in cardiac physiology. Another relevant mechanism, oxidative stress-related protein O-GlcNAcylation, was not directly detected in the present study. This is likely due to the fact that oxidative stress-associated protein regulation occurs upstream of and may precede detectable alterations in protein O-GlcNAcylation. Overall, these results identify candidate pathways linking prediabetic NDPK-B deficiency to early cardiac metabolic stress, providing a framework for future functional validation of O-GlcNAc-associated mitochondrial and cell-type-specific mechanisms.

It has been reported that excessive O-GlcNAcylation of mitochondrial proteins impairs oxidative phosphorylation, promotes mitochondrial fragmentation, and reduces ATP production, thereby compromising myocardial energetics in the diabetic heart [29,33,34]. This is supported by our proteomics data showing that the development of overt DCM is associated with distinct changes in the proteomic signature characterized by increased enrichment of lipid transmembrane transporter activity and very long-chain FA metabolic processes, alongside decreased enrichment of mitochondrial protein-containing complexes. Previous O-GlcNAc-based proteomic studies have similarly reported that chronic diabetes increases O-GlcNAc modifications on numerous mitochondrial and metabolic proteins in the heart, as well as on key regulators of myocardial contractility [26]. In addition, other studies have described altered O-GlcNAcylation of proteins involved in excitation-contraction coupling and calcium handling during the progression of DCM. In line with these reports, our findings highlight a prominent shift towards proteins involved in lipid transmembrane transporter activity. This observation reflects a hallmark of metabolic remodeling of the diabetic heart, which becomes pathologically dependent on FA uptake and utilization for energy production, often culminating in lipotoxicity [35,36,37]. The decreased enrichment of mitochondrial protein-containing complexes points to potential structural or functional remodeling within the mitochondria. Central components of key mitochondrial functions appear to be significantly affected, including protein synthesis (mitochondrial subunits MRPS and MRPL), protein import (TIMM50), and substrate entry into the Krebs cycle (PDHX). These alterations may compromise the mitochondrial capacity to sustain cardiac ATP production [38].

Taken together, our analysis of sWGA-enriched protein profiles reveals a potential shift from early metabolic/inflammatory disturbances and mitochondrial Complex I alterations toward wider lipid-driven metabolic remodeling and changes in mitochondrial component proteins as prediabetes advances to overt diabetes. Nevertheless, several limitations of the study should be acknowledged. While the STZ model reflects key DCM features, its systemic metabolic effects, such as weight loss, may interfere with the assessment of hypertrophic remodeling. Furthermore, using an sWGA-enriched proteome introduces the potential for capturing non-specific O-GlcNAcylated proteins, and co-enriched interacting proteins may also be enmeshed. Therefore, the candidates identified in this exploratory analysis are putative and require orthogonal validation. Future studies should employ site-specific glycoproteomics and functionally evaluate mitochondrial complex I activity, respiration, bioenergetics, and ROS generation.

## 4. Materials and Methods

### 4.1. Animals

All animal experiments conducted in this study were approved by the local ethics committee (G-165/20, G-157/25, Regierungspräsidium Karlsruhe, Germany) and performed in accordance with institutional guidelines (Medical Faculty Mannheim, Heidelberg University). The total animal sample size was determined based on established findings in the field and accumulated laboratory experience, in accordance with the 3Rs principle. NDPK-B^−/−^ mice were backcrossed with C57BL/6N mice for more than 10 generations to establish the C57BL/6N background [10]. Age-matched wild-type littermates served as controls. Mice were maintained under controlled environmental conditions at 21 °C and 55% relative humidity. Two to three animals were housed per medium-sized cage under a 12-h light/dark cycle with an illumination intensity of 150 lux. The animals were fed a standard maintenance diet (SSNIFF, Soest, Germany) and provided free access to tap water.

### 4.2. Induction of Diabetes

Type 1 diabetes mellitus was induced using STZ (Sigma-Aldrich, Taufkirchen, Germany). Animals aged 2.5 months were randomly divided into four experimental groups: WTNC (8 mice), WTDC (10 mice), NDPK-B^−/−^NC (8 mice), and NDPK-B^−/−^DC (10 mice). Animals with a body weight below 20 g were excluded from STZ treatment due to an increased risk of adverse effects. STZ was prepared immediately prior to use by dissolving it in cold citrate buffer (pH 4.5) to maintain stability and maximize biological activity. Following a 4-h fasting period, a single intraperitoneal injection of STZ was administered at a dose of 145 mg/kg body weight to both male homozygous NDPK-B^−/−^ mice and age-matched WT control mice. Potential confounding factors, including cage allocation and shelf position, treatment order, and animal handling, were minimized throughout the study. Mice were provided with tap water supplemented with 5% (*w*/*v*) glucose for the first 24 h post-injection. The successful induction of diabetes was verified two days after injection by measuring random blood glucose levels in tail blood (0.5 µL) with a portable blood glucose meter (ACCU-CHEK Guide, Roche, Mannheim, Germany). Mice with blood glucose concentrations exceeding 250 mg/dL were considered diabetic and included in subsequent analyses. Following diabetes induction, experimental groups were unblinded and animals were monitored regularly throughout the experimental period for changes in body weight, random blood glucose levels, and overall health status, including behavior, grooming, and food and water intake, to ensure animal welfare and to monitor disease progression.

### 4.3. Transthoracic Echocardiography

At three months following the induction of diabetes, cardiac structure and function were evaluated by transthoracic echocardiography in mice under light isoflurane anesthesia (CP Pharma, Burgdorf, Germany; induction with 3% isoflurane and maintenance with 1% isoflurane in 1 L/min oxygen), with spontaneous respiration monitored. Heart rates were maintained at approximately 400 bpm to prevent E/A wave fusion and ensure accurate diastolic assessment. To eliminate bias, all image acquisitions and subsequent offline analyses were performed in a blinded manner. During imaging, animals were placed in the supine position on a heating platform to maintain body temperature. Echocardiographic images were captured and analyzed using a Vevo 3100 high-resolution imaging system (FUJIFILM VisualSonics, Toronto, ON, Canada) equipped with a 30–40 MHz linear-array transducer (MX-550D, FUJIFILM VisualSonics, Toronto, ON, Canada). Further image processing and quantitative analyses were performed using the Vevo Lab Platform (version 5.5.0).

To comprehensively assess LV diastolic function, Doppler measurements were obtained from the apical four-chamber view under B-mode guidance. Pulsed-wave (PW) Doppler was applied to determine the mitral inflow velocities. With the sample volume positioned at the level of the mitral valve leaflet tips at the atrioventricular junction, peak early diastolic filling velocity (E wave) and late diastolic filling velocity during atrial contraction (A wave) were recorded. The E/A ratio was subsequently calculated. To assess the mitral annular myocardial velocities, tissue Doppler imaging (TDI) was performed in the apical four-chamber view. The sample volume was placed at the septal side of the mitral annulus to assess septal annular motion. Early diastolic annular velocity (E′) and late diastolic annular velocity during atrial contraction (A′) were measured, and the E′/A′ ratio was calculated. Heart rate was monitored throughout image acquisition, and Doppler recordings were carefully reviewed to ensure clear separation of the E and A waves and to exclude measurements affected by wave fusion. All measurements were averaged over 3–5 consecutive cardiac cycles to reduce beat-to-beat variability.

### 4.4. Tissue Collection

Body weight, blood glucose levels, and glycated hemoglobin (HbA1c, Infopia Clover A1c Analyser, EuroMedix, Cologne, Germany) were measured at the time of sacrifice, 3 months after diabetes induction. Following euthanasia, the heart was rapidly excised and immediately rinsed in physiological saline solution, gently dried using soft absorbent paper, and weighed. Subsequently, a transverse section from the mid-ventricular region was harvested for paraffin embedding and subsequent histological analysis. The LV was then dissected from the remaining cardiac tissue and reserved for further experimental analyses. TL was determined with a digital vernier caliper (Alpha Tools, Hermülheim, Germany) and subsequently applied as a reference parameter for normalization of cardiac measurements.

### 4.5. Protein Extraction and Immunoblotting

LV tissue was ground into a fine powder and resuspended in Radioimmunoprecipitation assay (RIPA) lysis buffer containing a protease inhibitor (cOmplete™ Protease Inhibitor Cocktail, Roche, Mannheim, Germany) and a phosphatase inhibitor (PhosSTOP™ phosphatase inhibitor tablets, Roche, Mannheim, Germany). The samples were then homogenized using a TissueLyser II (Qiagen, Hilden, Germany) at 300 Hz for four cycles of 30 s each, employing stainless steel beads (7 mm, Qiagen, Hilden, Germany). The TissueLyser adapter (Qiagen, Hilden, Germany) set was pre-cooled at −20 °C overnight before use to maintain a low temperature during sample homogenization. Protein lysates were denatured at 95 °C for 5 min with 1× Laemmli buffer containing glycerol.

Proteins (20 µg per lane) were loaded and separated on SDS-PAGE gels (consisting of a 4% stacking gel and a 6%, 10%, or 12% resolving gel, depending on the molecular weights of the target proteins) and subsequently transferred onto 0.2 µm nitrocellulose membranes using a wet transfer system at a constant voltage of 100 V for 1 h. After transfer, membranes were blocked for 1 h at room temperature using Roti-Block blocking solution (Carl Roth, Karlsruhe, Germany), followed by overnight incubation at 4 °C with the appropriate primary antibodies diluted in blocking solution. On the following day, membranes were washed three times with TBST (Tris-buffered saline containing 0.1% Tween-20, 15 min per wash) and subsequently incubated with the corresponding secondary antibodies for 1 h at room temperature. After washing, immunoreactive protein bands were detected using chemiluminescence substrates (Lumi-Light, Roche, Mannheim, Germany; or SuperSignal Femto, Thermo Scientific, Waltham, MA, USA) according to the manufacturers’ instructions. Chemiluminescent signals were captured using a Fusion FX imaging system (Vilber Lourmat, Eberhardzell, Germany). The following primary antibodies were employed in this study: mouse anti-O-GlcNAc (dilution 1:1000, ab2739, Abcam, Waltham, MA, USA); rabbit anti-GFAT1 (dilution 1:500, 14132-1-AP, Proteintech, Rosemont, IL, USA); rabbit anti-OGT (dilution 1:1000, O6264, Sigma-Aldrich, Taufkirchen, Germany); rabbit anti-OGA (dilution 1:1000, SAB4200267, Sigma-Aldrich, Taufkirchen, Germany); mouse anti-γ-Tubulin (dilution 1:3000, T6557, Sigma-Aldrich, Taufkirchen, Germany); rabbit anti-Fibronectin for the detection of total fibronectin (dilution 1:3000, F3648, Sigma-Aldrich, Taufkirchen, Germany). Corresponding secondary antibodies were as follows: goat anti-mouse IgG (H + L) (dilution 1:10,000, G21040, Invitrogen, Carlsbad, CA, USA); goat anti-rabbit IgG (H + L) (dilution 1:10,000, G21234, Invitrogen, Carlsbad, CA, USA).

### 4.6. Immunofluorescence Staining

To determine ventricular cardiomyocyte size, WGA staining was performed to delineate cardiomyocyte cell membranes. Formalin-fixed LV tissue samples were dehydrated, embedded in paraffin, and then sectioned (5 µm). The slides were deparaffinized in xylene and rehydrated through descending ethanol concentrations. Following overnight incubation with fluorescently labeled WGA (dilution 1:40, Sigma-Aldrich, Taufkirchen, Germany), the tissue sections were washed three times in PBS for 10 min each and finally mounted using ROTI^®^ Mount FluorCare mounting medium (Carl Roth, Karlsruhe, Germany). Fluorescence images were acquired using a microscope (Zeiss AXIO Scan.Z1, Carl Zeiss Microscopy, Jena, Germany) equipped with appropriate filters. Representative images were captured from both the subendocardial and subepicardial layers of the posterior wall of the LV. Cardiomyocyte cross-sectional area was quantified using ImageJ software Fiji Version v1.54p (National Institutes of Health, NIH, Bethesda, MD, USA) by manually tracing individual cardiomyocytes with clearly defined membranes.

### 4.7. Picrosirius Red Staining

Picrosirius Red staining was conducted according to the manufacturer’s instructions (Niepoetter, Bürstadt, Germany) to visualize and quantify collagen deposition. Briefly, paraffin sections were incubated in Picrosirius Red solution for 1 h at room temperature, allowing specific staining of fibrillar collagens. Collagen deposition was quantified using ImageJ software. Digital brightfield images of stained sections were acquired under identical microscope and camera settings for all samples. For analysis, color thresholding was applied to selectively identify Sirius Red-positive areas corresponding to collagen fibers. The collagen content was expressed as the percentage of the Sirius Red-positive area relative to the total tissue area. Measurements were performed on entire transverse heart sections as well as separately for the subendocardial and subepicardial layers of the LV myocardium.

### 4.8. Immunoprecipitation and LC-MS/MS Proteomic Analysis

To identify potential candidates associated with altered O-GlcNAcylation, an exploratory proteomics analysis was performed. LV tissue from non-diabetic and diabetic WT and NDPK-B^−/−^ mice was processed for enrichment of O-GlcNAc-modified proteins followed by mass spectrometry-based analysis. Cardiac tissue was homogenized in 240 µL ice-cold RIPA lysis buffer, and protein concentrations were determined using a BCA assay. For each sample, 0.5 mg of total protein was incubated with 30 µL of sWGA agarose beads to enrich O-GlcNAc-modified proteins. The samples were rotated gently for 24 h at 4 °C to allow binding. Subsequently, the beads were washed three times for 3 min each at 4 °C with buffer containing 50 mM Tris-HCl (pH 7.4), 100 mM NaCl, and 0.1% Triton X-100 to remove unbound proteins, and the enriched O-GlcNAc-containing protein fraction was recovered and subjected to LC-MS/MS analysis. Proteins were eluted in 100 µL urea-HEPES lysis buffer, heated at 56 °C for 20 min, cooled, and centrifuged at 25,000× *g* for 15 min at 4 °C. Proteins were reduced with 10 mM dithiothreitol (30 min, 37 °C), alkylated with 55 mM iodoacetamide (45 min, dark), and digested with sequencing-grade trypsin. Peptides were desalted and analyzed by nanoLC-MS/MS in Data-Independent Acquisition mode on an Astral mass spectrometer coupled to a Vanquish Neo nanoLC system (Thermo Fisher Scientific, Waltham, MA, USA). The ion-source voltage was set to 1.8 kV. MS1 spectra were acquired over a scan range of 380–980 *m*/*z* at a resolution of 240,000, with a maximum ion injection time of 5 ms and an automatic gain control (AGC) target of 500%. The 380–980 *m*/*z* range was divided into 300 consecutive isolation windows for continuous fragmentation and signal acquisition. Precursor ions were fragmented by higher-energy collisional dissociation using a normalized collision energy of 25. Fragment ions were detected in the Astral analyzer with a maximum ion injection time of 3 ms and an AGC target of 500%. Protein identification was performed by searching the acquired mass spectrometry data against the UniProt protein sequence database. The selected database served as the reference for peptide-spectrum matching and subsequent protein identification. The sample data were further processed by bioinformatic analysis using R (Version 4.4.3).

Proteins with fewer than three quantified data points were omitted from subsequent analyses. PCA was performed on log2-transformed, normalized label-free intensities from the O-GlcNAc immunoenriched cardiac proteomes of four groups. Volcano plots were generated for the indicated pairwise contrasts: WTDC versus WTNC, NDPK-B^−/−^NC versus WTNC, NDPK-B^−/−^DC versus WTDC, and NDPK-B^−/−^DC versus NDPK-B^−/−^NC. Fold changes were calculated as the ratio of group means (log2 scale), and proteins were considered differentially enriched at a nominal *p* < 0.05 and an absolute fold change ≥ 1.2 (|log2FC| ≥ 0.2630). This exploratory screening approach was chosen to maximize sensitivity for novel pathway discovery. Proteins meeting these thresholds were classified as showing increased or decreased enrichment. Pathway annotation was performed with Metascape Version 3.5 using a two-contrast intersection strategy. Proteins that were increased in WTDC relative to WTNC and those that were increased in NDPK-B^−/−^NC relative to WTNC were subjected to Gene Ontology (GO) over-representation analysis across the biological process (BP), cellular component (CC), and molecular function (MF) categories. GO terms significant in both comparisons were intersected to define WT-diabetic-elevated pathways. Top terms are shown as bar plots. Proteins belonging to consensus pathways were Z-scored by row and visualized as hierarchically clustered heat maps.

### 4.9. Statistical Analysis

All quantitative data are presented as mean ± SEM. Statistical analyses were performed using two-way ANOVA with Tukey’s post hoc test for multiple comparisons or Student’s *t*-test. All analyses were performed using GraphPad Prism version 8 (GraphPad Software, San Diego, CA, USA). *p* values < 0.05 were considered statistically significant.

## Data Availability

The data supporting the findings of this study are available from the corresponding author upon reasonable request. The mass spectrometry proteomics data have been deposited in the ProteomeXchange Consortium via the PRIDE partner repository with the dataset identifier PXD081812. The reviewer access token is “74EIBQgq6XDo”.

## Supplementary Materials

The following supporting information can be downloaded at: https://www.mdpi.com/article/10.3390/ijms27177518/s1.
