# Supplementary material for: Early Cardiomyopathy in Prediabetic NDPK-B-Deficient Mice Is Associated with Remodeling of the Mitochondrial O-GlcNAc Proteome
Source: Int J Mol Sci. 2026 Aug 22;27(17):7518. doi: 10.3390/ijms27177518 (PMC13566771; doi:10.3390/ijms27177518)
Supplement: Supplementary file 1 [file ijms-27-07518-s001.zip › figure and table legends.pdf]

Table S1: O-GlcNAc-associated proteins are differentially regulated in diabetic and prediabetic left ventricles. Proteomic analysis of left ventricular O-GlcNAcylated proteins identified numerous significantly regulated proteins in the following comparisons: (A) WTDC vs. WTNC, (B) NDPK-B$^{-/-}$NC vs. WTNC, (C) NDPK-B$^{-/-}$DC vs. WTDC, and (D) NDPK-B$^{-/-}$DC vs. NDPK-B$^{-/-}$NC.

Figure S1: Heart weight/body weight ratio remains comparable in diabetic animals. Quantification of heart weight/body weight ratio. n = 8-10.

Figure S2: Protein abundance of the O-GlcNAc cycle enzymes OGT and OGA remains unchanged in left ventricles. Protein abundance was determined by immunoblot analysis. Panels show the densitometric quantification of (A) OGT and (B) OGA. n = 5.

Figure S3: Shared O-GlcNAc-associated patterns identify common pathways in prediabetic and diabetic cardiomyopathy. sWGA-enriched proteomic profiles were compared to identify shared protein alterations between prediabetic and diabetic left ventricles. Proteins were selected based on their consistent regulation across both conditions relative to WTNC controls. (A) Heatmap illustrating the relative O-GlcNAcylation abundance of specific target proteins associated with the identified pathways. Colors denote row-scaled Z-scores (red: increased; blue: decreased). (B) Pathway enrichment analysis performed on the subset of proteins exhibiting these shared regulatory trends. Bar lengths indicate the significance of enriched terms, plotted as $-\log_{10}(p)$.
